# Unlocking Precise Lung Cancer Detection Through Minimal Panel Immunostaining in Small Biopsy Samples

**DOI:** 10.7759/cureus.63159

**Published:** 2024-06-25

**Authors:** Lakshmi Priya Asokan, Sumithra A, Vallal Kani, Chitra Srinivasan

**Affiliations:** 1 Department of Pathology, Saveetha Medical College and Hospitals, Saveetha Institute of Medical and Technical Sciences, Saveetha University, Chennai, IND

**Keywords:** differential diagnosis, small biopsies, histopathology, lung cancer, small cell carcinoma, adenocarcinoma, squamous cell carcinoma, immunohistochemistry

## Abstract

Introduction

Lung cancer diagnosis faces challenges due to morphological heterogeneity and limited biopsy tissue. This study evaluates the efficacy of a minimal panel immunostaining technique using immunohistochemical markers like napsin A, thyroid transcription factor 1 (TTF-1), p63, and synaptophysin to improve the precision of lung carcinoma subclassification.

Methods

A retrospective analytical study was conducted at the Histopathology Laboratory of Saveetha Medical College and Hospital, Chennai, from January 2018 to February 2024. A total of 64 lung carcinoma cases were analyzed. Inclusion criteria included biopsy samples from lung lesions with a confirmed diagnosis of lung carcinoma based on histomorphological examination, covering all age groups and both genders. Non-carcinomatous lung lesions were excluded. Clinical data were obtained from the Medical Information Archiving Software (MIAS) database and histopathological examination request forms. Under a light microscope, tissue samples were examined after being fixed in formalin, processed, and stained with hematoxylin and eosin (H&E). Additionally, a minimal panel of immunohistochemical markers, including napsin A, TTF-1, p63, and synaptophysin, was used to subclassify lung carcinomas.

Results

The age group older than 50 years was the most affected, with a higher incidence in males. Histologically, 49% of cases were adenocarcinoma, 42% were squamous cell carcinoma, and 9% were small cell carcinoma. Immunohistochemistry (IHC) results adjusted these proportions to 54.6% adenocarcinoma, 31.2% squamous cell carcinoma, and 14% small cell carcinoma, showing a 5.6% increase in adenocarcinoma cases. The most common adenocarcinoma pattern was mixed, followed by acinar. TTF-1 and napsin A were crucial for identifying adenocarcinoma, while p63 was key for squamous cell carcinoma. Synaptophysin confirmed neuroendocrine differentiation in small cell carcinoma.

Conclusion

Incorporating a minimal panel of IHC markers significantly enhances the accuracy of lung carcinoma subclassification, addressing diagnostic challenges posed by morphological heterogeneity and limited sample size. This approach supports more precise and efficient clinical care for patients with lung cancer. Further validation in diverse clinical settings is recommended.

## Introduction

Lung cancer remains one of the most significant contributors to cancer-related morbidity and mortality worldwide. It is a challenging illness with many subtypes that have unique molecular and histological characteristics [1]. Non-small-cell lung cancers (NSCLCs) account for about 85% of instances of lung cancer. Squamous cell carcinoma and adenocarcinoma are the two most common histological subtypes of non-small-cell lung malignancies [2]. The diagnosis of primary and metastatic lung cancers has become challenging in recent years. Pathologists are requested to evaluate smaller tissue samples because transbronchial and endobronchial biopsies are becoming increasingly important in the initial evaluation of lung abnormalities [3]. To prevent severe side effects and find the most efficient therapeutic options for the treatment of lung cancer, recent research has demonstrated the need for histopathological subtyping [4]. When a patient has advanced lung cancer and cannot undergo radical surgery, the histopathological diagnosis should be made using small biopsy samples obtained through transbronchial biopsy (TBB) or fine needle aspiration (FNA). These specimens are often crushed and lose morphology, making them unsuitable for histological diagnosis [2]. However, developments in immunohistochemistry (IHC) and molecular biology have expanded our comprehension of lung tumors [4]. It has been suggested that NSCLC subtypes can be distinguished using several immunohistochemical markers, such as napsin A and thyroid transcription factor 1 (TTF-1) in adenocarcinoma and tumor protein 63 (p63) and protein 40 (p40), which are frequently expressed in squamous cell carcinoma [5]. This study aims to determine the efficacy of IHC markers in accurately identifying different subtypes of lung carcinoma, including adenocarcinoma, squamous cell carcinoma, and small cell carcinoma.

## Materials and methods

This retrospective analytical study was conducted at the histopathology laboratory of our college from January 2019 to February 2024 after getting approval from the Institutional Review Board (IRB approval number: 160/04/2024/PG/SRB/SMCH). The study population includes samples from lung biopsies reported as lung carcinoma. A total of 64 lung carcinoma cases were analyzed for the study. The inclusion criteria were biopsy samples obtained from lung lesions with a confirmed diagnosis of lung carcinoma based on histomorphological examination and patients of all age groups and both genders. The exclusion criteria were made for cases of non-carcinomatous lung lesions. The clinical details, including age, gender, relevant history, and investigations regarding any cases, were taken from the MIAS (Medical Information Archiving Software) database of our hospital and the histopathological examination request forms. Microsoft Office Excel 2016 (Microsoft Corporation, Redmond, WA) was used to tabulate and correlate all pertinent data. To detect the malignancy, the collected tissue specimens were fixed in formalin, processed, stained with hematoxylin and eosin (H&E) stain, and examined through the microscope to evaluate the cellular morphology, tissue architecture, and malignant characteristics. Additionally, IHC was used to subclassify lung carcinomas. During the process of IHC, antigen retrieval is used to unmask antigens in tissue sections, which is essential for antibody binding. The next step is the application of the primary antibody, where specific antibodies are applied to bind to the target antigens in the tissue. Finally, the secondary antibody is applied. This antibody, often conjugated to a detection system, binds to the primary antibody. The bound antibodies were visualized using a chromogenic substrate, resulting in a color change at the sites of the antibody-antigen complexes. The results were evaluated to determine the diagnosis and categorization of lung carcinoma based on the staining pattern seen.

Data were entered in a Microsoft Excel sheet and statistical analysis was done using SPSS version 22.0 software (IBM Corp., Armonk, NY). Categorical variables were expressed as frequencies and percentages. A P-value of <0.05 was taken as statistical significance. The variables used included independent variables like pre-IHC histological classification (adenocarcinoma, squamous cell carcinoma, small cell carcinoma), post-IHC histological classification (adenocarcinoma, squamous cell carcinoma, small cell carcinoma), and IHC markers (TTF-1, napsin A, p63, synaptophysin) and dependent variables like change in the number of cases for each histological type pre- and post-IHC. The covariates were age, gender, and tumor site. The data analysis was done using descriptive statistics like frequency and percentage, and inferential statistics, including the chi-square test, sensitivity, specificity, positive predictive value (PPV), and negative predictive value (NPV).

The table below outlines the primary antibodies used in this study (Table 1).

**Table 1:** Details of primary antibodies used in the study. TTF-1: thyroid transcription factor 1; p63: tumor protein 63.

Antibody	Source	Clonality	Clone	Species	Dilution	Pre-treatment
TTF-1	PathnSitu	Monoclonal	HBPP	Rabbit	Pre-diluted	None
Napsin A	PathnSitu	Monoclonal	EP205	Rabbit	Pre-diluted	None
p63	PathnSitu	Polyclonal	4A4	Mouse	Pre-diluted	None

## Results

**Figure 1:**
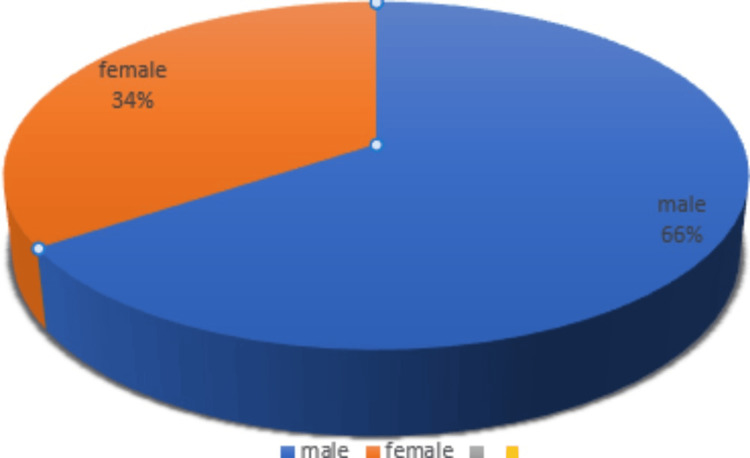
Gender distribution.

A total of 64 lung carcinoma cases were analyzed during the study period. All of them were biopsy specimens, obtained either through fibreoptic bronchoscopy or CT-guided biopsy. The age of the cases of NSCLC ranged from 46 to 83 years, with the mean age being 60 years. The number of males greatly outnumbered the females in the study. Out of 64 cases, 42 (65.6%) were males and 22 (34.4%) were females, as shown in Figure 1. The age distribution and gender were compared, and it was found that there was a male predominance noted across all the age groups. Among the 22 females, the most common age group was 61 to 70 years, followed by 51 to 60 years. In addition, of the 42 cases in the male population, 33 (78%) were older than 50 years, whereas only nine (22%) were below 50 years.

**Figure 2:**
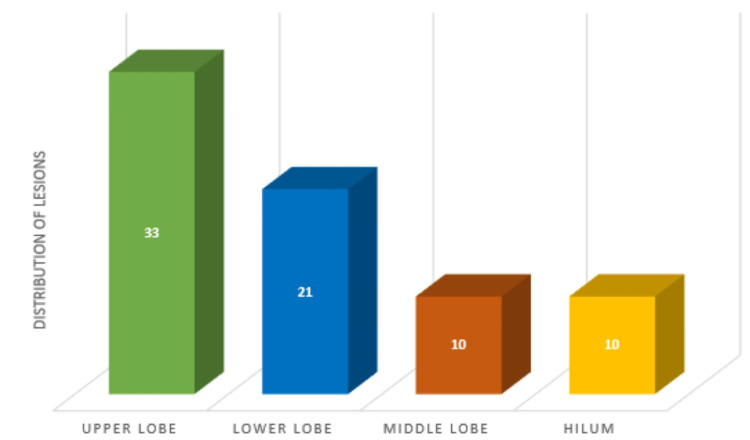
Distribution of tumors based on location.

In 40 cases (63%), the lesion was found in the left lung, while in 24 cases (37%), it was found in the right lung. In the majority of cases, the tumor was located in the upper lobe. Specifically, 52% (n = 33) of the lesions were in the upper lobe, 33% (n = 21) in the lower lobe, and 15% (n = 10) each in the hilum and the middle lobe, as shown in Figure 2.

**Figure 3:**
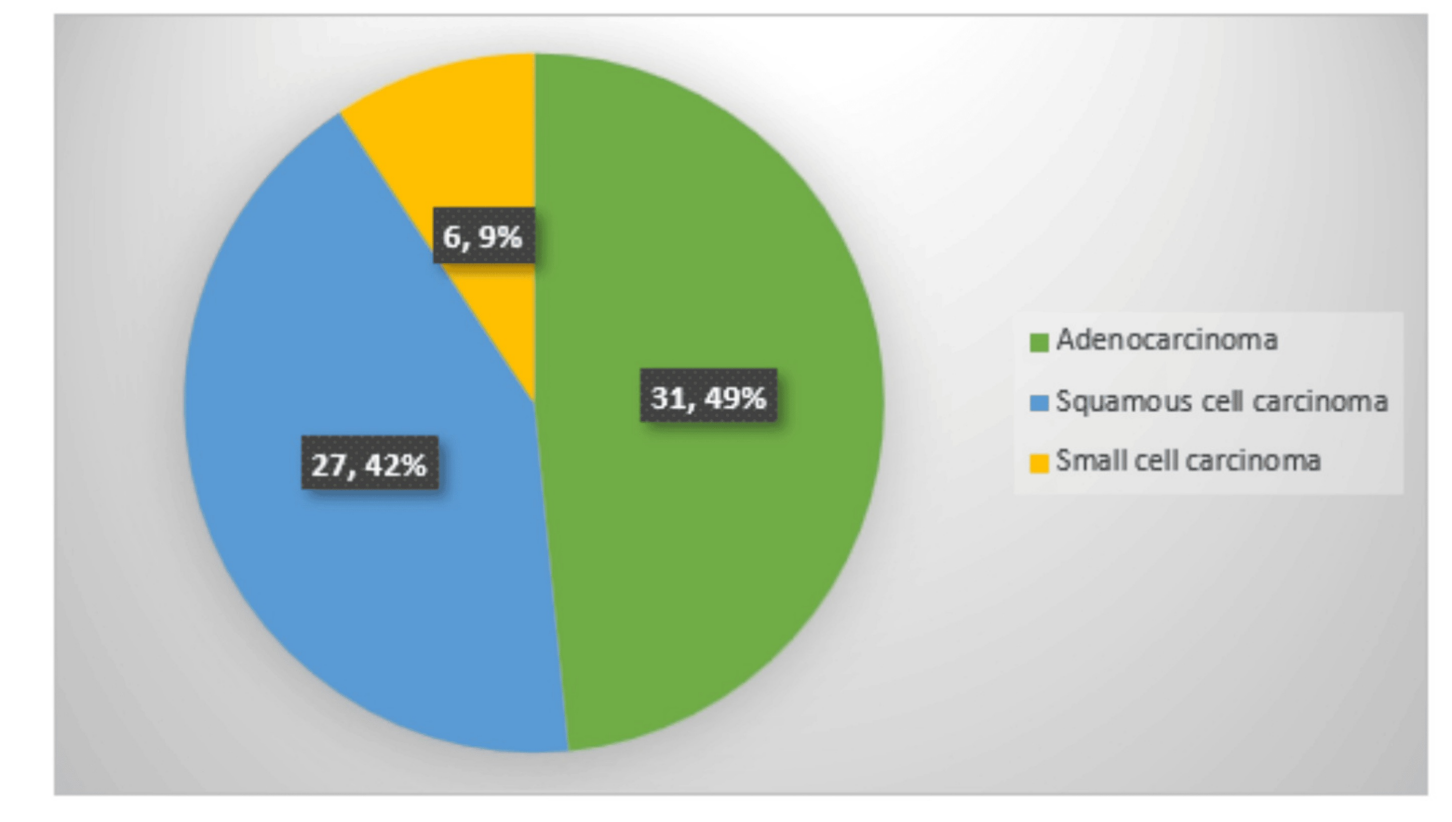
Distribution of lung tumors before using immunohistochemistry.

As per the records in the histopathology department, the 64 tumors had been initially reported histologically according to the WHO Classification of Tumors of the Lung, Pleura, Thymus and Heart, 4th edition. The most common histological type of lung carcinoma was adenocarcinoma (48.4%, n = 31), followed by squamous cell carcinoma (42%, n = 27) and small cell carcinoma (9%, n = 6) before using IHC, as shown in Figure 3.

NSCLC had the highest number of reported instances, with 31 cases of adenocarcinoma and 27 cases of squamous cell carcinoma, while six cases were small cell carcinoma. We identified different patterns in adenocarcinoma comprising acinar (7), solid with mucin production (3), lipidic pattern (2), papillary pattern (1), and mixed pattern (18), as shown in Table 2.

**Table 2:** Spectrum of lung carcinoma.

Type	Number of cases (n = 64)	(%)
Adenocarcinoma and its various patterns (n = 31)		48
Acinar pattern	7	22
Solid pattern with mucin production	3	10
Lipidic	2	6
Papillary pattern	1	3
Mixed pattern	18	58
Squamous cell carcinoma	27	42
Small cell carcinoma	6	9

On application of immunohistochemical stains, 31 cases exhibited positivity for both napsin A and TTF-1 while being negative for p63 and synaptophysin. p63 was solely positive in 20 cases, where the other stains were negative. Two instances exhibited positivity for napsin A and TTF-1 separately. Additionally, synaptophysin, a marker for neuroendocrine differentiation, was positive in nine cases, as shown in Table 3.

**Table 3:** IHC staining pattern in different types of lung tumors. IHC: immunohistochemistry; TTF-1: thyroid transcription factor 1; p63: tumor protein 63.

IHC done	n = 64	(%)
TTF-1+, Napsin A+, p63-, Synaptophysin-	31	48.1
TTF-1-, Napsin A-, p63+, Synaptophysin-	20	31.2
TTF-1+, Napsin A-, p63-, Synaptophysin-	2	3.1
TTF-1-, Napsin A+, p63-, Synaptophysin-	2	3.1
TTF-1-, Napsin A-, p63-, Synaptophysin+	9	14.5

The tumors were reclassified following immunohistochemical analysis involving napsin A, TTF-1, p63, and synaptophysin. Tumors exhibiting coarse granular cytoplasmic positivity for napsin A, nuclear positivity for TTF-1, and negative staining for p63 were diagnosed as adenocarcinomas. Adenocarcinoma was the predominant histological type observed in the majority of cases. Tumors demonstrating strong nuclear positivity for p63 and negative staining for TTF-1 and napsin A were categorized as squamous cell carcinoma. Those exhibiting strong cytoplasmic staining were classified as small cell carcinoma, as shown in Table 4.

**Table 4:** The frequency distribution of the histological type of lung tumors after IHC. IHC: immunohistochemistry.

Histological type after IHC	No. of cases	Percentage
Adenocarcinoma	35	54.6%
Squamous cell carcinoma	20	31.2%
Small cell carcinoma	9	14%
Total number of cases	64	100%

Following immunohistochemistry, adenocarcinomas continued to be the predominant type. Their count increased from 31 to 35 cases, while squamous cell carcinoma decreased from 27 to 20 cases, and small cell carcinoma increased from six to nine cases, out of a total of 64 cases, as shown in Table 5.

**Table 5:** Significance of adding immunohistochemistry in the diagnosis of subtypes of lung cancer (n = 64). Chi-square statistic: 1.285; p-value: 0.04 (statistically significant). IHC: immunohistochemistry; TTF-1: thyroid transcription factor 1; p63: tumor protein 63.

Lung carcinoma subtypes	Histomorphological diagnosis (N = 64) (%)	After using IHC (TTF-1, napsin A, p63, and synaptophysin)	Subtype characterization
Adenocarcinoma	31 (49%)	35 (54.6%)	Increment by 5.6%
Squamous cell carcinoma	27 (42%)	20 (31.2%)	Reduction by 10.8%
Small cell carcinoma	6 (9%)	9 (14%)	Increment by 5%

A chi-square test was done to compare the distribution of histological types before and after IHC. The chi-square statistic was 1.285 and the p-value was 0.04, which is considered statistically significant. This suggests that there is a statistically significant difference in the distribution of histological types before and after IHC.

Table 6 provides a comprehensive view of the diagnostic performance of IHC in subclassifying lung carcinomas, showing high specificity and PPV across all types, with varying levels of sensitivity and NPV.

**Table 6:** Sensitivity, specificity, PPV, and NPV of IHC of lung carcinomas. PPV: positive predictive value; NPV: negative predictive value; IHC: immunohistochemistry.

Lung carcinoma subtypes	Sensitivity (%)	Specificity (%)	PPV (%)	NPV (%)
Adenocarcinoma	88.6	100	100	87.9
Squamous cell carcinoma	74.1	100	100	84.1
Small cell carcinoma	66.7	100	100	94.8

**Figure 4:**
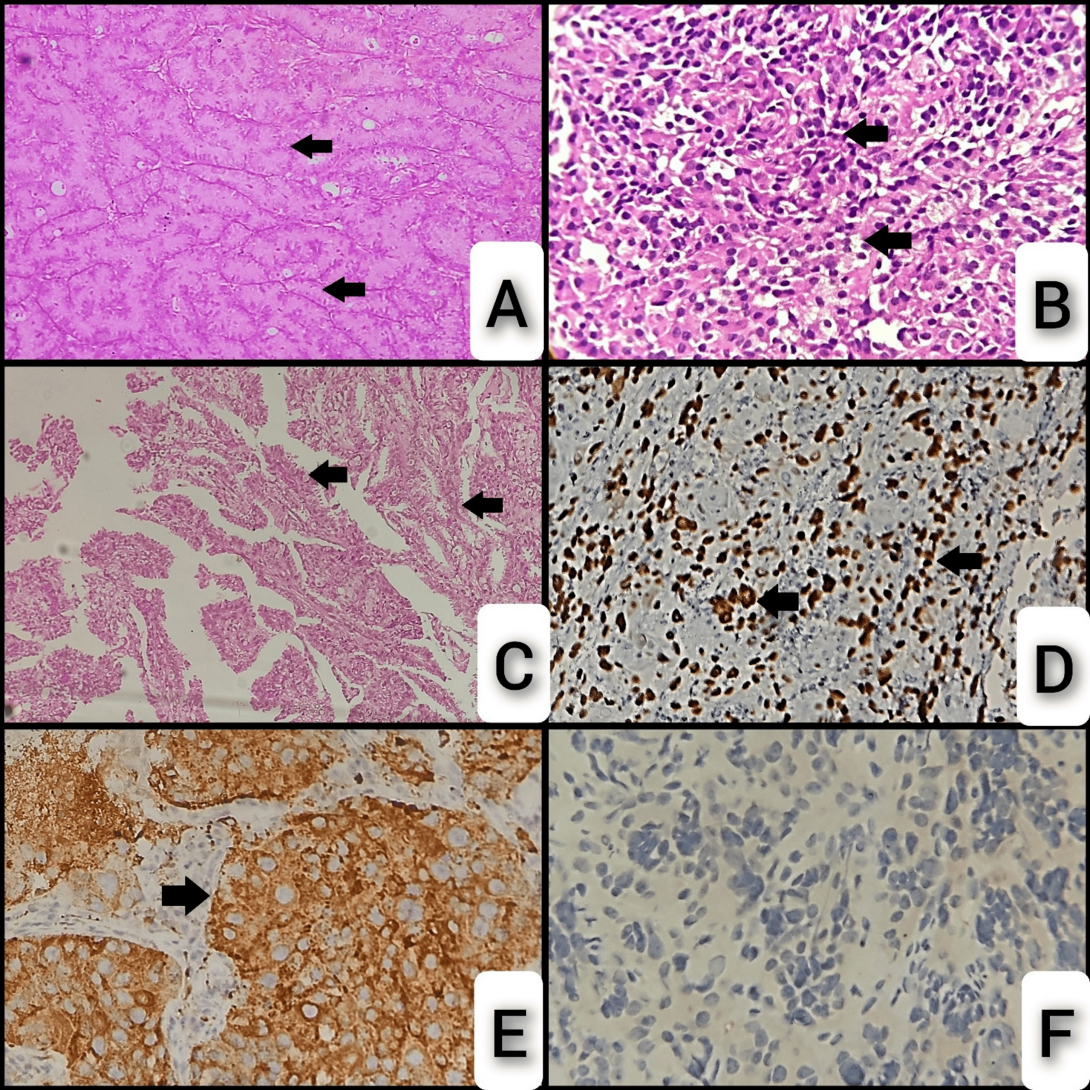
Hematoxylin & eosin section and IHC panel of adenocarcinoma. A: Section showing a neoplasm composed of tumor cells arranged in closely packed glands (arrows). B: Section showing a neoplasm composed of a moderate amount of eosinophilic cytoplasm and mildly pleomorphic hyperchromatic nuclei arranged in sheets and vague glands (arrows). C: Section showing a neoplasm composed of tumor cells arranged in a papillary pattern (arrows). D: IHC of TTF-1 shows strong nuclear positivity in tumor cells (arrows). E: IHC of napsin A shows strong granular cytoplasmic staining of tumor cells (arrow). F: IHC of p63 shows negative staining of tumor cells. IHC: immunohistochemistry; TTF-1: thyroid transcription factor 1; p63: tumor protein 63.

Figure 4 shows H&E sections displaying various patterns and malignant characteristics, and the IHC panel depicting adenocarcinoma.

**Figure 5:**
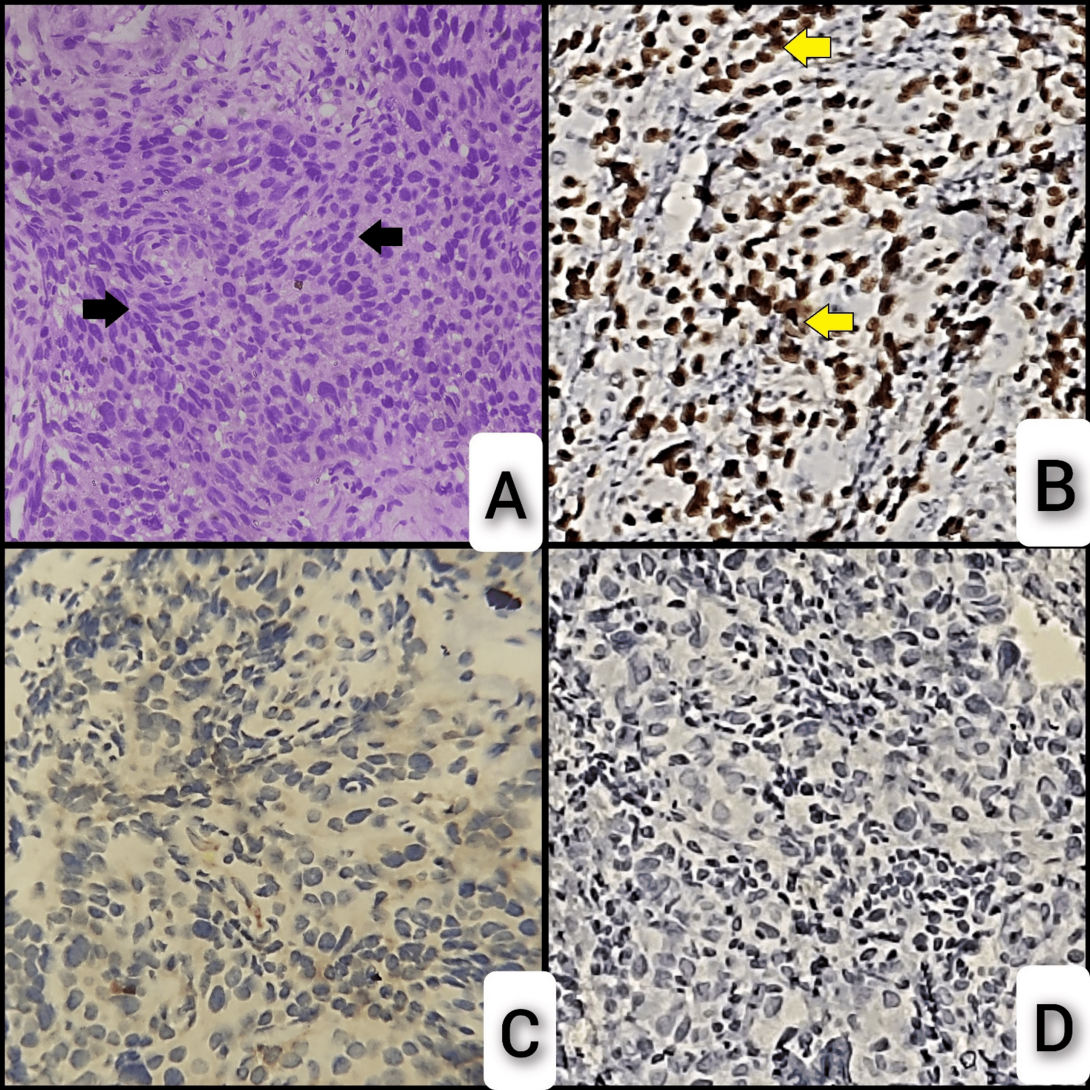
Hematoxylin & eosin section and IHC panel of squamous cell carcinoma. A: Section shows a malignant neoplasm composed of cells having pleomorphic, hyperchromatic nuclei with scant cytoplasm arranged in sheets and nests (arrows). B: IHC of p63 shows strong nuclear positivity in tumor cells (arrows). C: IHC of TTF-1 shows negative staining of tumor cells. D: IHC of napsin A shows negative staining of tumor cells. IHC: immunohistochemistry; TTF-1: thyroid transcription factor 1; p63: tumor protein 63.

Figure 5 shows H&E sections displaying malignant characteristics and the IHC panel of squamous cell carcinoma.

**Figure 6:**
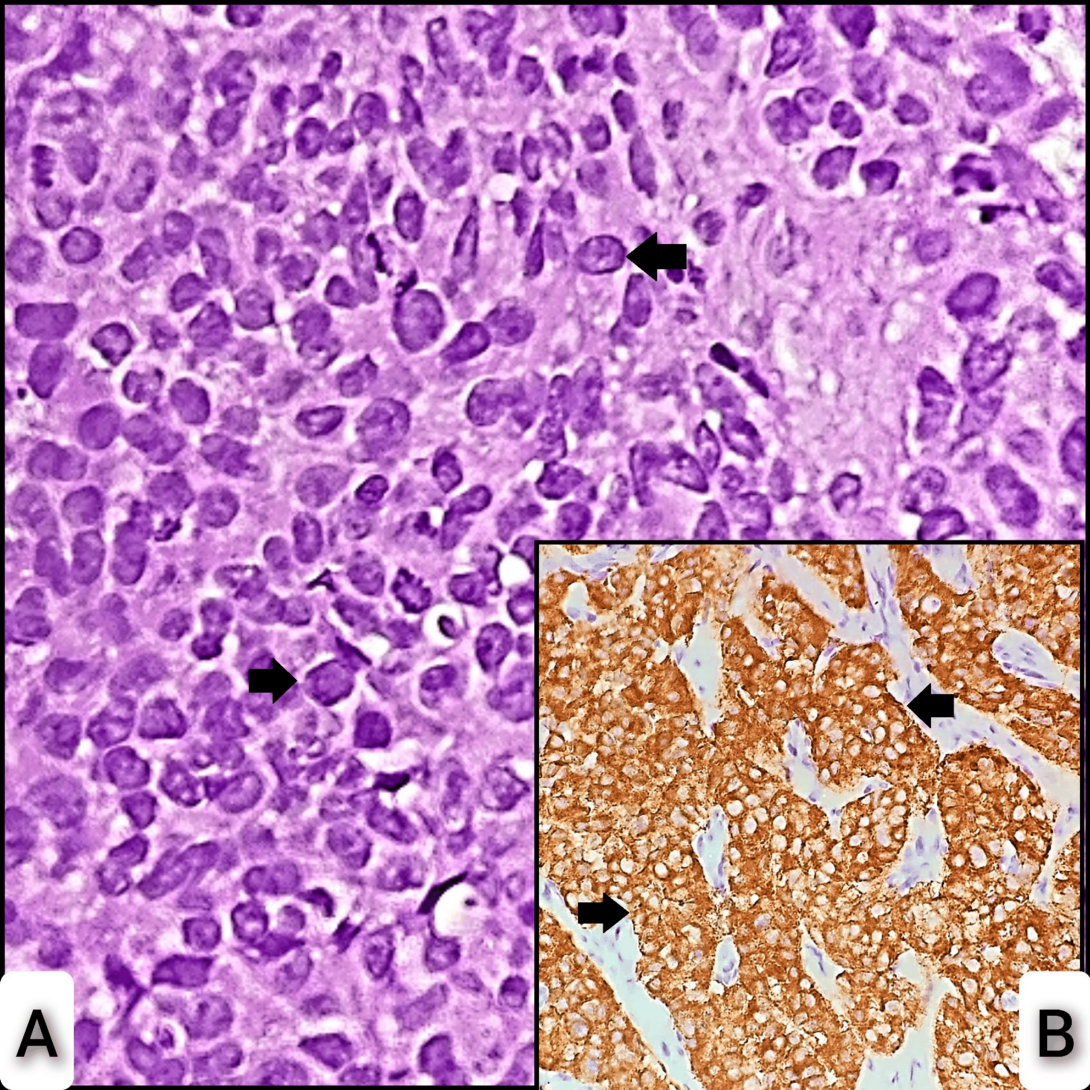
Hematoxylin & eosin section of small cell carcinoma and IHC of synaptophysin marker. A: Section shows a neoplasm composed of cells having a moderate amount of eosinophilic cytoplasm and moderately pleomorphic round nuclei with dispersed chromatin arranged in sheets (arrows). B: IHC of synaptophysin shows strong cytoplasmic positivity in tumor cells (arrows). IHC: immunohistochemistry.

Figure 6 shows H&E sections displaying malignant characteristics and neuroendocrine marker (synaptophysin) of small cell carcinoma.

## Discussion

Lung cancer is a major health concern due to its association with increased cancer-related deaths. Around 85% of lung cancer cases are NSCLC, the most prevalent type of epithelial lung carcinoma [2]. The WHO 2021 report for thoracic tumors emphasizes IHC, genetic testing, and biomarkers over morphology in classifying tumors. Accurate classification of tumors is necessary for early diagnosis and the implementation of advanced treatment strategies, including targeted and combination therapy [6]. Diagnostic samples are limited, as the accuracy in staging and histological classification of advanced lung cancers is poor, given that most lung cancers are unresectable at the time of diagnosis [7]. This thereby focuses on the importance of making accurate diagnoses, including histological type and molecular profiling, even in small biopsy and cytology samples [8].

In cases of well-differentiated or moderately differentiated tumors, morphological evaluation alone is enough to classify them. In cases of poorly differentiated carcinomas, heterogenous tumors like adenosquamous tumors, and inadequate diagnostic material, arriving at a diagnosis with morphology alone is often challenging. In such cases, the use of an appropriate IHC panel will aid in arriving at an accurate diagnosis and precise typing of lung tumors. Our study aims to use a minimal IHC panel to reduce the usage of the term NSCLC-NOS (not otherwise specified) and specifically classify these tumors.

In our study, the most common age group that presented with lung carcinoma was above 50 years of age, and males were more commonly affected among all age groups, and this was in concordance with Stojsic et al. [9].

Among the cases studied, we histologically classified lung carcinoma according to the WHO classification as follows: adenocarcinoma was 48.2%, squamous cell carcinoma was 31.2%, and small cell carcinoma was 14%. The most common subtype in our study turned out to be adenocarcinoma, which was consistent with Mukhopadhyay et al. [10]. As mentioned in Table 1, we analyzed the various patterns in adenocarcinoma and found the common pattern to be a mixed type, followed by an acinar pattern. We encountered a case of adenocarcinoma with a papillary pattern, which turned out to be TTF-1 and p63-positive.

We used a panel of four IHCs: napsin A, TTF-1, p63, and synaptophysin. TTF-1 is expressed in a variety of ways during lung development, ranging from early respiratory diverticulum morphogenesis to its greater expression toward the lung's periphery as the more distant airways emerge. TTF-1 exhibits positive staining in cases of adenocarcinoma; however, its expression is seen to decline inversely with tumor differentiation, i.e., with lesser expression in poorly differentiated tumors [10,11]. Napsin A is considered a novel marker for lung adenocarcinoma. It is a proteinase that is associated with the maturation of surfactant protein B [12,13]. Tumor protein 63 (p63) is a well-established marker for squamous cell carcinoma. It is a transcription factor belonging to the family of p53 genes, which is found on chromosome 3q28 [14]. Expression of p63 is seen in non-neoplastic lung tissue, like basal reserve cells of stratified squamous epithelium and glandular epithelium [15,16]. Synaptophysin, an integral membrane glycoprotein, is seen in presynaptic neurosecretory vesicles. This is utilized for recognizing lung tumors that exhibit neuroendocrine differentiation [17,18].

We observed the following outcomes of using IHC: adenocarcinoma (54.6%), squamous cell carcinoma (31.2%), and small cell carcinoma (14%). After that, there is an increment of about 5.6% and 5% in the number of cases of adenocarcinoma and small cell carcinoma. We found that this increase in the number of cases of adenocarcinoma was initially reported as NSCLC, which was the same in other studies [19,20]. It was observed that the reason for these discrepancies was due to challenges in identification due to morphologically heterogeneous features like solid, poorly differentiated types, and a very limited representative tumor area. NSCLC favoring adenocarcinoma is a morphological diagnosis that needs further classification using IHC. Likewise, cases of poorly differentiated squamous cell carcinoma pose diagnostic difficulty by morphology alone. Some studies showed that a panel of IHC was beneficial to rule out cases of the squamoid variant of adenocarcinoma [21,22].

It is seen that the majority of NSCLC cases were subclassified by TTF-1 and p63, with the requirement of an additional marker, napsin A, in only a few cases. To determine the line of differentiation, TTF-1 and p63 should be included in the diagnostic panels, according to the previous studies.

In our study, we did not encounter double positivity with TTF-1 and p63, but the literature suggests that focal p63 coexpression can be seen in cases of adenocarcinoma. In such cases, the expression of tumor protein (p63) in a diffusely positive TTF-1 tumor should be supportive of adenocarcinoma [10,23,24]. It is important to note that this positivity of p63 and TTF-1 should be seen in the same population to be regarded as adenocarcinoma, while if it is in different populations, it is interpreted as adenosquamous. Notably, in a study, it was found that the preferential reactivity of p63 in TTF-1-positive adenocarcinoma was seen to have EML4-ALK translocation, implying that this expression might not be merely aberrant [25].

Similarly, interpretation of TTF-1 and p63 double negative cases is challenging, and in such cases, a more specific marker like napsin comes to aid. Also, it is observed in the literature that consistent positivity for p63 is observed in squamous cell carcinoma of the lung, and hence the double negative cases can be supportive of squamous cell carcinoma [10,26,27].

In the context of neuroendocrine tumors, IHC is not essential for the diagnosis of small-cell carcinoma. Nevertheless, we utilized synaptophysin to validate neuroendocrine characteristics and to rule out the possibility of small cell variants of squamous cell carcinoma, lymphoma, and other blue round cell tumors. However, caution should be exercised when interpreting immunohistochemical stains, particularly in cases of significantly crushed biopsies with otherwise difficult-to-interpret morphology [28].

We observed a 5% increase in the incidence of small cell carcinoma following the use of IHC. All the small-cell carcinoma cases were stained with synaptophysin, a specific neuroendocrine marker. We have not encountered large-cell neuroendocrine carcinoma (LCNEC) in our study. Owing to the difficulty in identifying the pattern and the limited sample available for IHC, the diagnosis of LCNEC on a small biopsy is onerous.

Therefore, by applying a limited panel of IHC, our study showed a considerable improvement in the subclassification of lung cancer. This is seen in agreement with a few other studies [7,23].

Limitations

The limitations of this study are limited sample size, tumor heterogenicity, follow-up, and long-term outcome.

## Conclusions

This study underscores the importance of incorporating IHC into the diagnostic workflow for lung carcinoma, particularly in cases where morphology alone may be insufficient. By leveraging appropriate IHC panels, clinicians can enhance the accuracy of tumor subclassification, thereby enabling more tailored and effective treatment strategies for patients with NSCLC.
